# Carbon Dot-Linked Hydrogel-Composite Scaffold with Sequential Release of Multi-Drug for Bone Repair

**DOI:** 10.3390/gels12060471

**Published:** 2026-05-29

**Authors:** Beibei Wang, Xuetong Sun, Hao Sun, Jiacheng Yu

**Affiliations:** 1 College of Life Sciences, Zhuhai College of Science and Technology, Zhuhai 519040, China; 2 College of Chemistry, Jilin University, Changchun 130012, China

**Keywords:** bone regeneration, antibacterial activity, carbon dots, multi-drug, sequential release

## Abstract

Bone repair is a complex and dynamic process that demands implanted scaffolds to provide temporal-specific functions: antibacterial activity in the early stage, followed by angiogenic and osteogenic stimulation in later stages. This study introduces a biomimetic scaffold composed of a filled Gel-OSA hydrogel and a 3D-printed PLA framework, enabling sequential multi-drug release for bone regeneration. Zero-dimensional arginine-derived carbon dots were incorporated into the hydrogel to achieve rapid release after implantation, conferring potent antibacterial activity and ROS regulation. Meanwhile, chondroitin sulfate (CS)-loaded mesoporous bioactive glass nanoparticles were immobilized onto the 3D-printed PLA surface via a polydopamine coating, allowing sustained release of CS and Ca/P ions to enhance the scaffold’s long-term osteoinductive capability. The composite scaffold further demonstrated combined effects in promoting cell proliferation and osteogenic differentiation in vitro. Collectively, these findings suggest that this biomimetic scaffold, designed for temporally controlled multi-drug release, represents a promising therapeutic strategy for the reconstruction of bone tissue.

## 1. Introduction

Large bone defects resulting from resection, trauma, or age-related diseases present significant clinical challenges [1,2,3]. In recent years, tissue engineering scaffolds have emerged as a promising alternative to conventional bone grafts for defect repair [4,5]. Bone healing, however, is a complex, sequential process involving inflammatory, reparative, and remodeling phases, each requiring precise temporal regulation of bioactive molecules [6,7]. An ideal scaffold should therefore be capable of stage-specific release of therapeutic factors to match the dynamic healing progression.

Biodegradable polymers such as polylactic acid (PLA)—an FDA-approved aliphatic polyester with tunable degradation and mechanical properties—are widely used as scaffold materials [1,8]. Nevertheless, PLA alone lacks intrinsic bioactivity and exhibits limited cell affinity, which can hinder cellular integration and osteogenic differentiation [9,10]. In contrast, hydrogels offer a three-dimensional, hydrophilic network that mimics the native extracellular matrix (ECM) and can serve as carriers for growth factors and drugs [11,12]. By combining these two classes of materials with distinct degradation profiles, it is possible to preserve their individual advantages while achieving temporally controlled release.

Early-stage bacterial infection remains a major concern following implantation, often necessitating extensive surgical intervention and prolonged antibiotic therapy. Although systemic antibiotics are commonly used, their overuse risks inducing bacterial resistance [13,14,15]. Carbon dots, zero-dimensional nanomaterials with excellent water dispersibility, low toxicity, and multifunctional bioactivity, have recently attracted attention in regenerative medicine [16,17]. Arginine-derived carbon dots (Arg-CDs) in particular possess a moderate positive surface charge that disrupts bacterial membranes, leading to effective microbial inactivation [18,19,20]. Moreover, Arg-CDs can modulate intracellular reactive oxygen species (ROS) levels, upregulate antioxidant enzymes in mammalian cells, and exert anti-inflammatory effects [21], thereby protecting host tissues while targeting pathogens.

CS, a key glycosaminoglycan in bone and cartilage ECM, plays vital roles in cell migration, proteoglycan synthesis, and modulation of inflammatory responses during early bone regeneration. When loaded into mesoporous bioactive glass (MBG) nanoparticles, CS can be released in a sustained manner. MBG itself offers a high specific surface area, large pore volume, and the ability to release osteogenic ions (e.g., Si^4+^, Ca^2+^) [22,23,24], thereby enhancing collagen I deposition, alkaline phosphatase (ALP) activity, and biomineralization [25,26].

**Figure 1:**
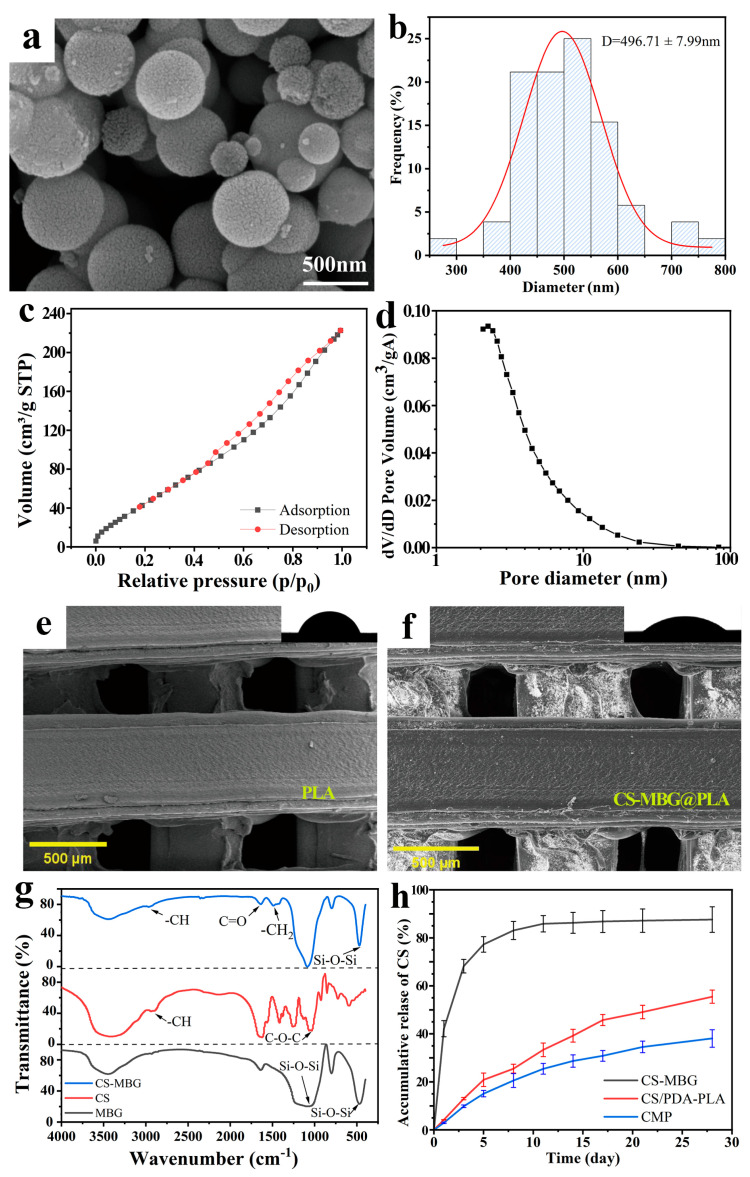
(**a**) SEM image and (**b**) particle size analysis of MBG particles. (**c**) N_2_ adsorption−desorption isotherms and (**d**) the pore diameter distribution of MBG particles. (**e**) SEM images of PLA and (**f**) CMP scaffolds. (**g**) FT−IR spectra of CS−MBG. (**h**) CS release from CS-MBG particles and CS−PLA and CMP scaffolds in PBS solution.

In this work, we designed a composite scaffold that integrates rapid Arg-CD release for early-stage antibacterial and immunomodulatory effects with sustained CS-MBG delivery for later osteogenesis. Arg-CDs were synthesized hydrothermally and incorporated into a Gel-OSA (GO) hydrogel crosslinked via Schiff base formation, enabling rapid release in the acidic microenvironment of an infected bone defect. A 3D-printed PLA scaffold was fabricated by fused deposition modeling (FDM) to provide structural support. By leveraging the strong adhesive properties of PDA, CS-loaded MBG nanoparticles were immobilized onto the PLA surface to ensure prolonged CS release. As illustrated in Figure 1, Arg-CDs are rapidly released upon implantation to provide immediate antibacterial activity and modulate macrophage polarization toward a pro-healing phenotype. Meanwhile, MBG nanoparticles enable efficient loading of CS and facilitate its controlled release in later stages, imparting sustained osteogenic activity to the scaffold. Experimental results confirm that this multi-drug release system effectively addresses stage-specific demands of bone regeneration. This cost-effective, easily fabricated multifunctional scaffold demonstrates considerable potential for advanced bone tissue engineering applications.

In this work, we report a composite scaffold that achieves sequential release of Arg-CDs and CS-MBG drugs, corresponding to stage-specific bone regeneration. Arg-CDs were synthesized hydrothermally and incorporated into a Gel-OSA hydrogel crosslinked via dynamic imine and borate ester bonds, enabling rapid release in the acidic microenvironment of an infected bone defect. A 3D-printed PLA scaffold was fabricated by fused deposition modeling (FDM) to provide structural support, and CS-loaded MBG nanoparticles were immobilized onto the PLA surface via a polydopamine coating to ensure prolonged CS release and bioactive ionic dissolution for long-term osteogenesis. The introduction of Arg-CDs at an optimized concentration precisely satisfies the early-stage antibacterial and anti-inflammatory demands—including potent bactericidal action against both Gram-positive and Gram-negative pathogens, intracellular ROS scavenging, and macrophage repolarization toward an M2 phenotype—while the composite assembly with CS-MBG simultaneously enhances the long-term osteoinductive capacity. Preliminary experimental results suggest that this multi-drug release system may effectively address stage-specific demands of bone regeneration. This cost-effective and easily fabricated multifunctional scaffold shows promise for potential applications in advanced bone tissue engineering.

## 2. Results and Discussion

### 2.1. Characterization of CS-MBG Nanoparticles

The relatively loose surface morphology imparted by the mesoporous nature of the MBG nanoparticles was examined by SEM (Figure 1a). Particle size analysis indicated an average diameter of 496.71 ± 7.99 nm (Figure 1b). Nitrogen adsorption–desorption measurements were performed to further characterize the mesoporous structure. As shown in Figure 1c, the isotherm exhibited a type-IV profile with an H4-type hysteresis loop across the relative pressure (p/p_0_) range of 0.0–1.0. Pore size distribution analysis (Figure 1d) revealed that pore diameters were primarily concentrated between 2 and 10 nm, with a most probable pore diameter of 2.25 nm and an average pore diameter of 6.12 nm, confirming a micro–mesoporous structure. This structure contributed to a high specific surface area of 223.68 m^2^/g, providing abundant active sites and substantial drug-loading capacity.

On the macroscopic scale, the PLA scaffold exhibited a well-defined porous structure with a porosity of 45.80%, satisfying the structural prerequisites for cell ingrowth and angiogenesis in bone tissue engineering. Figure 1e,f present the SEM images of PLA and CMP scaffolds. The pristine PLA surface appeared relatively smooth, whereas the CMP scaffold exhibited a distinctly rougher morphology.

The surface microstructure alteration, coupled with the abundant amino and hydroxyl groups introduced by the PDA coating, collectively enhanced the surface hydrophilicity of the scaffold. As confirmed by the water contact angle (WCA) measurements (insets, Figure 1e,f), the CMP scaffold exhibited a lower WCA (39.59 ± 1.19°) compared to pristine PLA (71.47 ± 1.24°). This enhancement of wettability is conducive to promoting cell adhesion and spreading, thereby improving the overall cellular affinity of the scaffold.

FT-IR analysis of CS-doped MBG nanoparticles (Figure 1g) showed characteristic stretching vibrations of hydroxyl (–OH) and amide (–NH–) groups from CS in the 3200–3500 cm^−1^ region. Compared with pure CS, these peaks appeared broader and less intense, suggesting the formation of hydrogen bonds between the –OH/–NH groups of CS and the surface silanol (Si–OH) groups of MBG, which restricted vibrational freedom. At 1086 cm^−1^, the overlapping C–O–C stretching vibrations from the CS sugar ring and the Si–O–Si asymmetric stretching vibrations of MBG resulted in a pronounced increase in peak intensity, indicating enhanced molecular interaction. Furthermore, the nearly identical spectral profiles of MBG-CS and pristine MBG around 470 cm^−1^ confirm that the Si–O–Si inorganic framework of MBG remained intact after CS loading. The retention of saturated C-H stretching vibrations at 2968 cm^−1^ in the MBG-CS spectrum also confirms the preservation of CS’s organic structure, collectively supporting the stable immobilization of CS on the MBG surface through combined hydrogen bonding and electrostatic interactions.

The standard curve of CS was determined using a UV absorption spectrometer, and the CS loading content in the resulting nanoparticles was determined to be 9.61%. The release profile of CS from different groups—CS-MBG nanoparticles and CS-PLA and CMP scaffolds—was evaluated under simulated physiological conditions (PBS, pH 7.4, 37 °C). As shown in Figure 1h, the CS-MBG nanoparticle group displayed a release behavior characteristic of typical mesoporous carriers: CS, primarily adsorbed within and at the entrances of the MBG mesopores, was rapidly released following the complete medium penetration, achieving a high cumulative release of 83.08 ± 3.76% by day 8. In contrast, the CS-PLA group exhibited a cumulative release of 20.86 ± 2.78% by day 5; this release behavior is likely governed jointly by concentration gradient-driven diffusion and desorption-limited kinetics arising from multivalent interactions between CS chains and the PDA network. Notably, the CMP scaffold demonstrated a superior and sustained controlled-release performance. Its cumulative release reached 38.09 ± 3.66% by day 28, exhibiting a smooth and prolonged profile. This enhancement is attributed to the PDA coating, which acts as an effective diffusion barrier [27]. This barrier effectively restricts medium infiltration and retards drug diffusion, thereby significantly reducing the initial burst release while ensuring prolonged scaffold stability and sustained therapeutic efficacy.

### 2.2. Characterization of Arg-CDs

**Figure 2:**
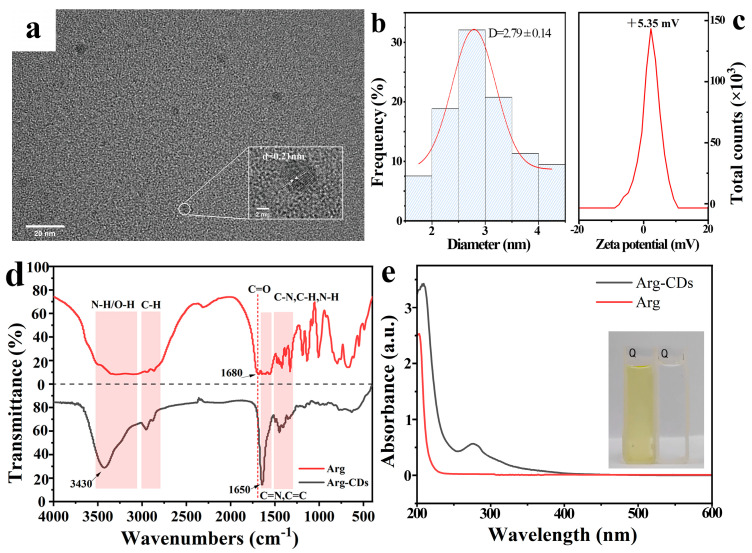
(**a**) TEM image of Arg−CDs. (**b**) Particle size analysis of Arg−CDs. (**c**) Zeta potential of Arg−CDs at pH 7.4. (**d**) FT−IR spectra of arginine and Arg−CDs. (**e**) UV−Vis absorption spectra and color under white light of arginine and Arg−CDs.

The Arg-CDs were synthesized using a straightforward hydrothermal method. TEM analysis revealed that the as-prepared Arg-CDs possessed a spherical morphology and were well-dispersed without significant aggregation (Figure 2a). The particle sizes fell within a narrow range of 1.5 to 4.5 nm (Figure 2b). High-resolution TEM images showed well-defined lattice fringes with an interplanar spacing of 0.21 nm, which is characteristic of the (100) crystal planes of graphitic carbon. Surface charge analysis indicated that the Arg-CDs exhibited a zeta potential of +5.35 mV at physiological pH (7.4) (Figure 2c). This net positive surface charge is likely attributable to the presence of exposed amino and residual guanidinium groups from the arginine precursor. Such a positive surface establishes a favorable electrostatic interface, which is crucial for interacting with negatively charged bacterial membranes and contributes to the enhanced antibacterial efficacy [28,29].

The surface chemistry of the Arg-CDs was further probed by FT-IR spectroscopy (Figure 2d). The spectrum displayed characteristic absorption bands at 1680 cm^−1^, 1640 cm^−1^, and 1450 cm^−1^, corresponding to the stretching vibrations of amide I (C=O), amide II (N–H), and amide III (C–N), respectively. The broad bands centered at 3430 cm^−1^ indicate the presence of –OH and –NH_2_ groups. These spectral features confirm that the synthesized CDs retain key functional groups derived from the arginine precursor, supporting their successful functionalization. UV-Vis spectroscopic analysis (Figure 2e) showed a distinct absorption peak at approximately 276 nm, which is assigned to the n-π* transition of carbonyl (C=O) and imine (C=N) chromophores. An additional peak around 210 nm corresponds to the π–π* transition of aromatic C=C bonds. Collectively, the FT-IR and UV-Vis data verify the successful synthesis of arginine-functionalized carbon dots with the intended chemical structure.

### 2.3. Characterization of GACP Composite Scaffold

**Figure 3:**
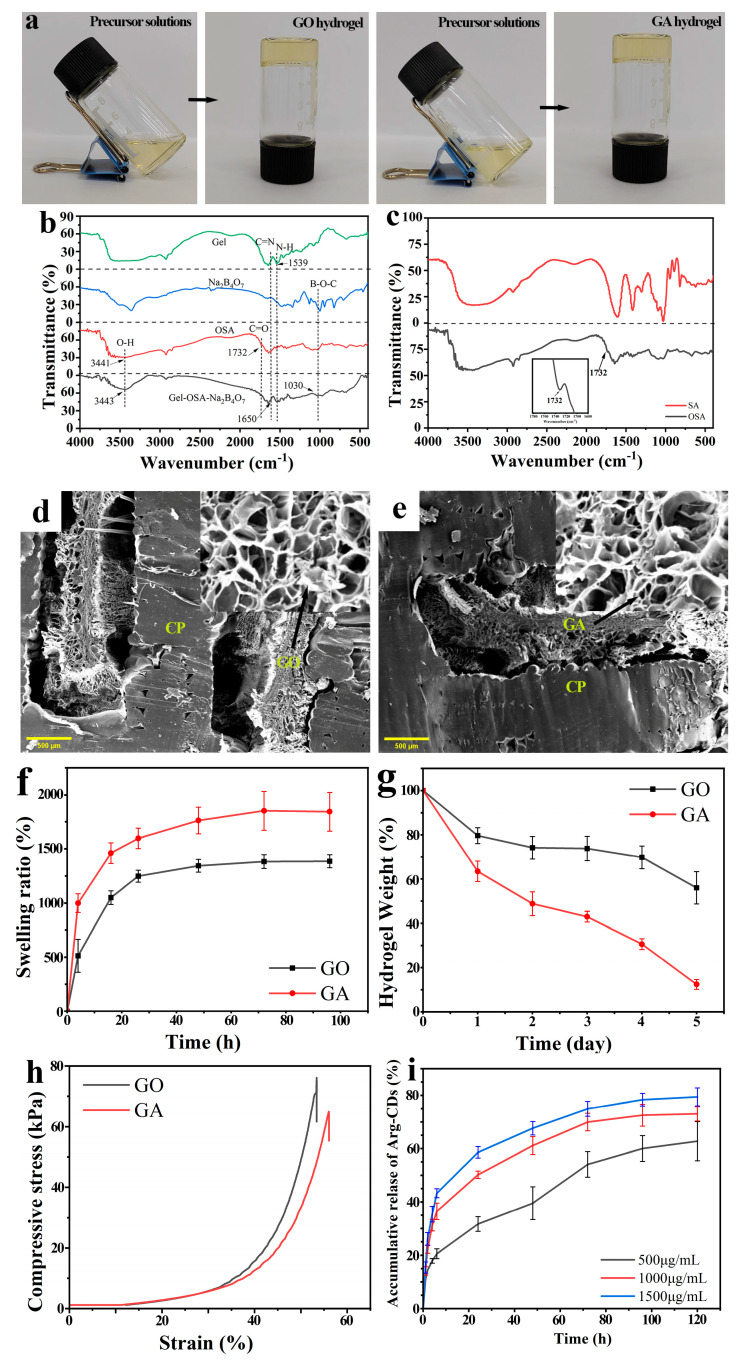
(**a**) Gelation process of GO and GA. (**b**,**c**) FT−IR spectra of GO hydrogel. SEM images of (**d**) GO and (**e**) GA scaffolds. (**f**) Swelling and (**g**) degradation curve of GO and GA. (**h**) Compressive stress–strain curve of GO and GA. (**i**) Arg−CD release from GA with different carbon point loads in PBS solution.

The GA hydrogel was prepared via homogeneous mixing of the Arg-CD suspension with solutions of OSA and gelatin/borax under physiological conditions (Figure 3a). The GO hydrogel network is constructed through two types of covalent crosslinks: imine bonds and borate ester bonds. OSA was synthesized by periodate oxidation of sodium alginate, achieving an oxidation degree of 51.48% as quantified by hydroxylamine hydrochloride titration.

FTIR analysis verified the successful synthesis of the GO hydrogel (Figure 3b). The characteristic stretching vibration of the aldehyde group (–C=O) from OSA appeared at 1732 cm^−1^ (Figure 3c), indicating that sodium alginate had been effectively oxidized by NaIO_4_. The broadening and reduced intensity of the –OH absorption band at 3441 cm^−1^ indicated interaction with anionic borate species. The presence of an amide I band at 1650 cm^−1^ is consistent with imine bond formation. Furthermore, a new peak at 1030 cm^−1^, corresponding to the B-O-C stretching vibration, confirmed the formation of borate ester bonds. Lyophilized samples of both GO and GA hydrogels displayed a uniform micro-scale structure (Figure 3d,e). Comparative analysis revealed that the GA hydrogel exhibited a faster degradation rate and a higher swelling ratio than the GO hydrogel (Figure 3f,g).

This can be attributed to the binding of Arg-CDs to the aldehyde groups on OSA, which reduces the effective Schiff base crosslinking density between gelatin and OSA. Consequently, the GA hydrogel showed a lower compressive modulus compared to the GO hydrogel (Figure 3h).

The release profile of Arg-CDs from GA hydrogels containing different concentrations of Arg-CDs was evaluated. As shown in Figure 3i, the cumulative release of Arg-CDs from GA at 5 days was 62.76 ± 7.35%, 73.05 ± 2.72% and 79.41 ± 3.34%, corresponding to Arg-CD loading concentrations of 500 μg/mL, 1000 μg/mL and 1500 μg/mL, respectively. All three concentration groups exhibit a biphasic release profile: an initial rapid “burst release” phase within the first ~24 h, followed by a slower, gradual release phase that remains ongoing up to 120 h, with no strict plateau observed. The cumulative release percentage of Arg-CDs is positively correlated with the initial loading concentration: higher initial concentrations of Arg-CDs result in a faster release rate, a higher burst release fraction, and a greater total cumulative release over the 120 h period.

### 2.4. In Vitro Antibacterial Ability of Composite Scaffolds

**Figure 4:**
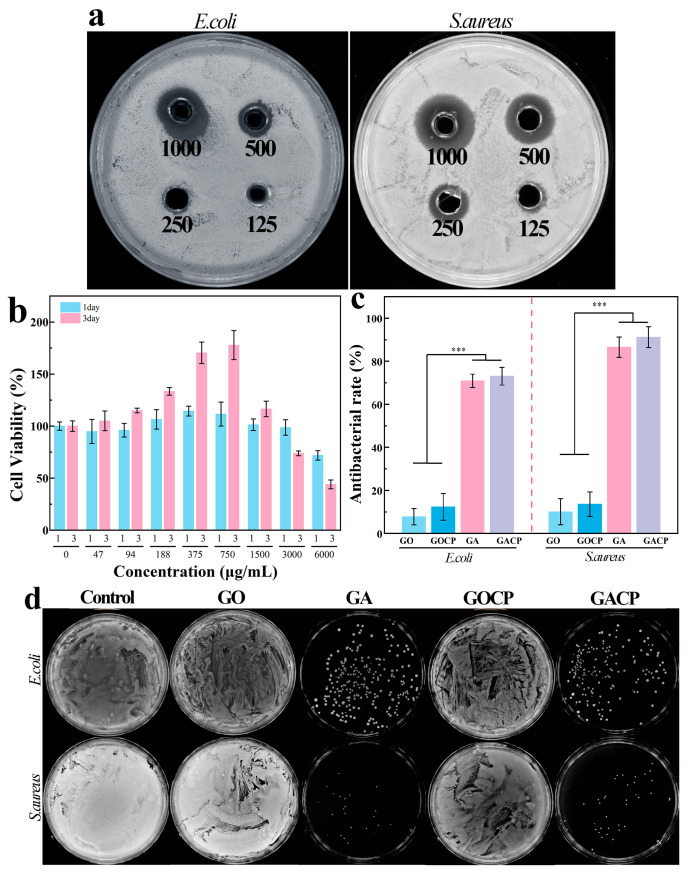
(**a**) Inhibition zones of the different concentrations of Arg-CDs in contact with *E. coli* and *S. aureus* (μg/mL). (**b**) Proliferation ability of MC3T3-E1 was measured by CCK-8 after 1 and 3 days of incubation in the Arg-CD solution. (**c**) Statistical graphs of the antibacterial rates of the scaffolds in each group after the scaffolds were cultured with *E. coli* and *S. aureus*, respectively, for 24 h. (**d**) Colony photos of *E. coli* and *S. aureus*, respectively, after culturing with the scaffolds for 24 h (*** *p* < 0.001 by Student’s *t* test).

Antimicrobial efficacy of drugs is typically concentration-dependent, yet supraphysiological concentrations may elicit cytotoxicity despite achieving effective bacterial clearance. *S. aureus* and *E. coli* were employed as representative Gram-positive and Gram-negative bacteria, respectively. Analysis of inhibition zone diameters (Figure 4a) showed that the antibacterial effect of the Arg-CD solutions against both strains was concentration-dependent, with larger zones corresponding to higher Arg-CD concentrations.

To determine the appropriate concentration of Arg-CDs, the minimum inhibitory concentration (MIC) against each strain was determined at pH 7.4 using the broth microdilution method. The MIC values for *E. coli* and *S. Aureus* were 103.6 and 198.7 μg/mL, consistent with previously reported findings. Meanwhile, the cytotoxicity of GA hydrogels loaded with varying concentrations of Arg-CDs was assessed using the CCK-8 assay. As shown in Figure 4b, cell proliferation ceased within one day in groups loaded with Arg-CDs at concentrations ≥6000 μg/mL, and within three days in groups with concentrations ≥3000 μg/mL. Notably, GA hydrogels loaded with Arg-CDs at 350–1000 μg/mL significantly promoted cell proliferation compared to other groups. Based on the MIC results, CCK-8 data, and the release profiles of hydrogels with different Arg-CD loadings, an intermediate concentration of 1000 μg/mL was selected for further scaffold fabrication.

The antibacterial performance of the composite scaffolds was further evaluated. As shown in Figure 4d, bacterial culture plates for the GO, GOCP, and control groups exhibited dense colonization by *S. aureus* and *E. coli*, whereas significantly fewer bacterial colonies were observed in the GA and GACP groups. Quantitative analysis based on optical density (OD) measurements of bacterial suspensions (Figure 4c) revealed that the antibacterial rates of GO and GOCP against both strains were below 20%, indicating negligible intrinsic antibacterial activity.

In contrast, the GA and GACP scaffolds showed pronounced antibacterial effects, with rates of 91.17 ± 1.85% against *S. aureus* and 73.00 ± 1.12% against *E. coli* after 24 h. As confirmed by zeta potential measurements (Figure 3c), Arg-CDs possess a moderately positive surface charge of +5.35 mV at physiological pH, which is conferred by abundant surface-exposed –NH_2_ groups. Since bacterial membranes are negatively charged and the biofilm microenvironment is often slightly acidic, Arg-CDs preferentially interact with bacteria over mammalian cells via electrostatic attraction. This interaction can disrupt the bacterial membrane and interfere with critical metabolic processes. Moreover, it has been reported that Arg-CDs can elevate intracellular ROS levels in bacteria and simultaneously suppress antioxidant enzyme expression [30], thereby impairing the bacterial capacity to scavenge ROS and leading to excessive oxidative stress. Overall, the combined effect of membrane disruption and ROS-mediated oxidative stress is considered to contribute to the potent bactericidal activity of Arg-CDs.

### 2.5. In Vitro Anti-Inflammatory Effects of Composite Scaffolds

Inflammatory responses, commonly triggered during the early phase of bone implantation, can significantly impede tissue regeneration. This stage is characterized by elevated intracellular ROS levels and polarization of RAW 264.7 macrophages toward a pro-inflammatory M1 phenotype [31], which may lead to excessive or prolonged inflammation. M1 macrophages secrete inflammatory cytokines such as TNF-α, IL-6, and IL-1β, which can reduce the recruitment of angiogenic cells. Uncontrolled inflammation not only delays healing but also creates a microenvironment unfavorable for osteogenesis.

**Figure 5:**
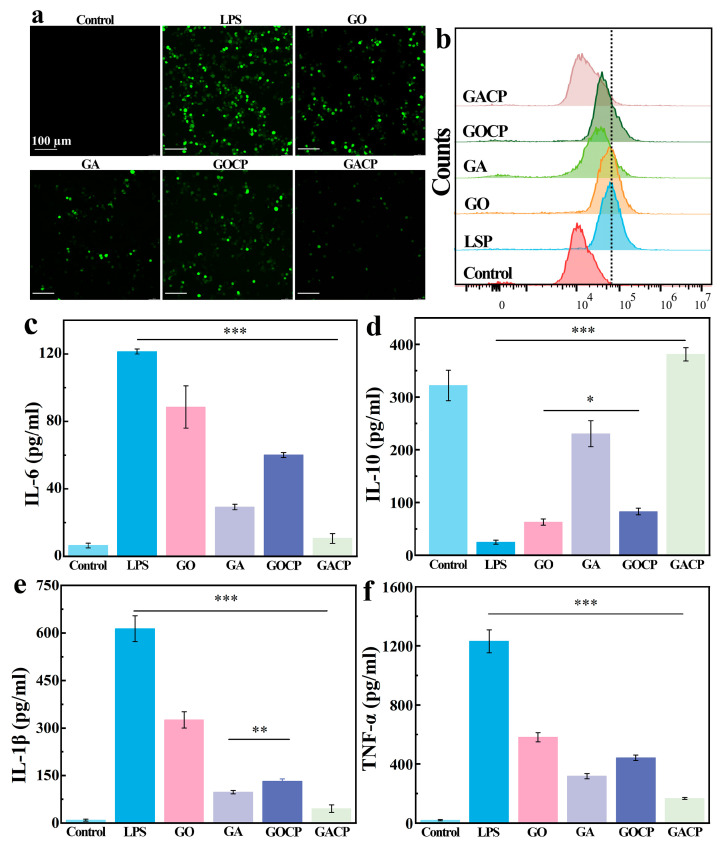
(**a**) Quantification of ROS levels in RAW264.7 cells as measured by the fluorescence intensity of DCFH-DA. (**b**) Flow cytometry analysis of the scale of ROS-positive cells. (**c**–**f**) The levels of IL-6, IL-10, IL-1β and TNF-α in the supernatant of RAW264.7 cells after various treatments (* *p* < 0.05, ** *p* < 0.01, *** *p* < 0.001 by Student’s *t* test).

To evaluate the anti-inflammatory potential of the scaffolds, RAW264.7 macrophages were stimulated with LPS to establish an inflammatory model. Intracellular ROS levels were detected using the fluorescent probe DCFH-DA. The LPS-treated group exhibited strong green fluorescence, indicating high ROS production. The fluorescence intensity was only slightly reduced in the GO and GOCP groups, suggesting limited ROS-scavenging capacity. In contrast, the GA and GACP groups showed a marked decrease in fluorescence signal (Figure 5a). This decrease can be attributed to Arg-CDs, which have been reported to upregulate key antioxidant enzymes such as superoxide dismutase and catalase, thereby effectively mitigating oxidative stress. Flow cytometry analysis further confirmed a significantly lower percentage of ROS-positive cells in the GA and GACP groups compared to the LPS group (Figure 5b), quantitatively validating their antioxidant capacity.

The secretion of inflammatory cytokines (IL-6, IL-1β, TNF-α) and the anti-inflammatory cytokine IL-10 was also quantified (Figure 5c–f). As expected, LPS stimulation significantly increased the release of TNF-α, IL-1β, and IL-6, while suppressing IL-10 production. In contrast, treatment with extracts from the GA and GACP groups—particularly GACP—substantially reduced the levels of pro-inflammatory cytokines and markedly increased IL-10 secretion. The GO and GOCP groups showed minimal modulatory effects, maintaining a pro-inflammatory profile. This favorable immunomodulatory effect is likely mediated by Arg-CDs, which appear to suppress the production of pro-inflammatory cytokines such as IL-1β, IL-6, and TNF-α while promoting IL-10 expression, thereby suggesting a potential shift in macrophage polarization toward an anti-inflammatory M2 phenotype [32]. To further assess the temporal persistence of the anti-inflammatory effect, ROS levels and cytokine secretion were measured at day 5 as well (see Figure S1). Consistent with the 24 h findings, the results at day 5 revealed no significant attenuation of the anti-inflammatory response, suggesting sustained efficacy over this time frame. Collectively, these results demonstrate that Arg-CDs confer significant ROS-scavenging and anti-inflammatory properties to the composite scaffolds, with the GACP scaffold exhibiting the most pronounced immunomodulatory efficacy.

### 2.6. In Vitro Biocompatibility and Osteogenic Properties

**Figure 6:**
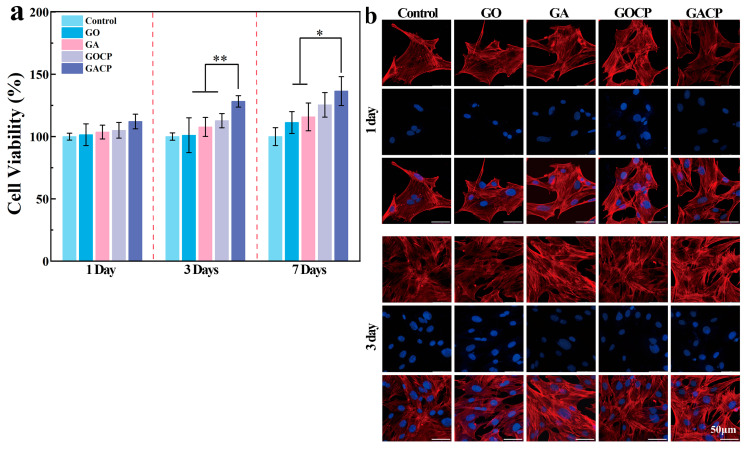
(**a**) Proliferation ability of MC3T3-E1 was measured by CCK-8 after 1, 3, and 7 days of incubation in the extract of the scaffolds. (**b**) Confocal images of MC3T3-E1 cultured in the extract of the scaffolds for 1 and 3 days; the cytoskeleton was treated with rhodamine–phalloidin (red), while the nucleus was treated with DAPI (blue) (* *p* < 0.05, ** *p* < 0.01 by Student’s *t* test).

The biocompatibility, viability and proliferation of MC3T3-E1 cells were assessed using the CCK-8 assay at different time points. Cell viability remained above 96% in all groups after 1, 3, and 7 days of culture (Figure 6a), confirming that the composite scaffolds are essentially non-cytotoxic and biocompatible at the tested concentrations, with no acute cytotoxicity, although sublethal effects have not been fully evaluated. Furthermore, the GACP composite scaffold significantly promoted cell proliferation compared to the other groups, suggesting its potential to actively support tissue regeneration.

Cell morphology was further evaluated using DAPI–phalloidin staining after 24 and 72 h of culture in scaffold extracts (Figure 6b). MC3T3-E1 cells in all groups displayed normal elongated morphology and well-spread cytoskeletal structures, demonstrating that the addition of Arg-CDs did not compromise the biocompatibility of the composite scaffolds. Compared to the control group, cells cultured in extracts from the GACP scaffold group exhibited significantly enhanced spreading and more developed actin filaments, indicating improved cell–substrate interactions and superior biocompatibility.

**Figure 7:**
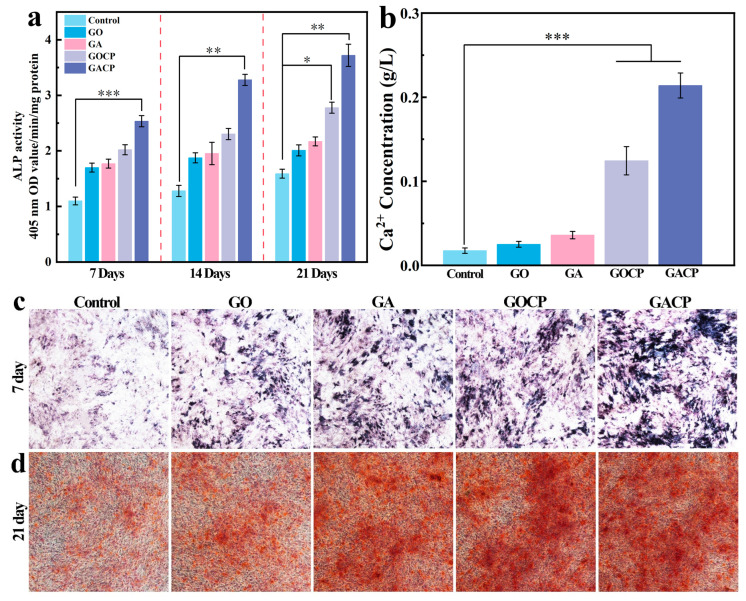
(**a**) ALP activity of MC3T3-E1 cultured in the extract of composite scaffolds after 7, 14 and 21 days of osteogenic differentiation. (**b**) Calcium deposition quantification of MC3T3-E1 seeded in the extract of composite scaffolds after 21 days of osteogenic differentiation. (**c**) ALP staining of MC3T3-E1 seeded in the extract of composite scaffolds. (**d**) Alizarin red staining of MC3T3-E1 seeded in the extract of composite scaffolds (* *p* < 0.05, ** *p* < 0.01, *** *p* < 0.001 by Student’s *t* test).

Alkaline phosphatase (ALP) activity serves as an early marker of osteogenic differentiation, while mineralized nodule formation—an indicator of mature osteoblast function—can be assessed using alizarin red S (ARS) staining [33]. Throughout the culture period, ALP activity increased in all groups relative to the control group. Notably, on days 7, 14, and 21, ALP activity in the GA group was slightly higher than that in the GO group, indicating the excellent osteoinductive activity of Arg-CDs (Figure 7a). ALP staining indicates that the GACP composite scaffold significantly upregulated the expression of ALP compared to the other groups (Figure 7c). After 21 days, a large number of calcium nodules were observed in the GA, GOCP and GACP groups (Figure 7d). In contrast, less calcium deposition was produced in the GO group. Quantitative analyses of mineralized nodule formation (Figure 7b) further confirmed statistically significant enhancements in the GACP group compared with all other groups. It is acknowledged that the present study is limited to in vitro evaluations, and the lack of in vivo experiments represents a major limitation. Animal studies are required to assess the scaffold’s performance under physiological conditions. Such investigations are planned for future work.

## 3. Conclusions

In this study, we successfully fabricated a biomimetic macro/micro/nanoporous scaffold (Arg-CDs@OSA-Gel/CS-MBG@PLA) for the temporally controlled co-delivery of Arg-CDs and CS-MBG to promote bone regeneration. Arg-CDs were rapidly released during the initial stage and demonstrated potent antibacterial activity, efficient scavenging of intracellular ROS, inhibition of pro-inflammatory cytokine release, and promotion of anti-inflammatory cytokine release—key processes in mitigating oxidative stress and resolving inflammation. Meanwhile, CS-MBG was immobilized on the PLA surface via a PDA-assisted composite interface, enabling sustained release of osteogenic signals. Owing to the combination of favorable antibacterial properties and bone-forming capacity, the GACP composite scaffold exhibited a positive effect on osteogenic differentiation. Thus, Arg-CDs, as a zero-dimensional nanomaterial, endow the bone scaffold with both antibacterial and osteoinductive activities, potentially offering insights for biomaterial-based tissue regeneration.

## 4. Materials and Methods

### 4.1. Materials and Reagents

Gelatin (Gel), sodium alginate (SA; M:G ratio = 1.2:1, molecular weight range 1.0 × 10^5^–1.5 × 10^5^), sodium tetraborate, sodium periodate, and L-arginine (Arg) were purchased from Aladdin (Shanghai, China). Chondroitin sulfate (CS, bovine origin) was acquired from Dibai (Shanghai, China). Ammonium hydroxide, tetraethyl orthosilicate (TEOS), hexadecyltrimethylammonium bromide (CTAB), and triethyl phosphate (TEP) were also obtained from Aladdin.

Dopamine hydrochloride was sourced from Sigma-Aldrich (St. Louis, MO, USA). The 4% paraformaldehyde, 1 M Tris-HCl buffer (pH 8.0), and phosphate-buffered saline (PBS, pH 7.4) were supplied by Biosharp (Hefei, China). Lipopolysaccharide (LPS) and the Enhanced BCA Protein Assay Kit, Alkaline Phosphatase Assay Kit, Calcein/PI Cell Viability/Cytotoxicity Assay Kit, and BCIP/NBT Alkaline Phosphatase Color Development Kit were purchased from Beyotime (Shanghai, China).

The MC3T3-E1 and RAW264.7 cell lines were obtained from the Cell Bank of the Chinese Academy of Sciences (Shanghai, China). Dimethyl sulfoxide (DMSO) was provided by Solarbio (Beijing, China). Fetal bovine serum (FBS), α-Minimum Essential Medium (α-MEM), and Dulbecco’s Modified Eagle’s Medium (DMEM) were purchased from Procell (Wuhan, China). Osteogenic induction medium was acquired from Oricell (Guangzhou, China). Enzyme-linked immunosorbent assay (ELISA) kits from Ruixin Biotech (Quanzhou, China) were used to quantify the concentrations of pro-inflammatory cytokines (IL-1β, IL-6, and TNF-α) and the anti-inflammatory cytokine IL-10.

### 4.2. Construction of PLA Scaffolds

The PLA scaffolds, with a pore size of 500 μm and a porosity of 50%, were fabricated using an FDM 3D printer (Creatbot F430, Henan Suwei Electronic Technology, Zhengzhou, China). The PLA filament was supplied by Caige (Zhuhai, China). Scaffold models were designed with Free CAD (Version 1.1) software. The printing parameters were set as follows: nozzle diameter 0.75 mm, infill density 80%, and layer height 0.6 mm. All printed scaffolds exhibited a cubic geometry with dimensions of 11 mm (length) × 7 mm (width) × 5 mm (height). After printing, the scaffolds were vacuum-dried for 24 h before further use.

### 4.3. Fabrication of CS-MBG/PLA Scaffolds

MBG nanoparticles were synthesized according to an established sol–gel protocol as previously described [34]. To load CS into the MBG, 0.1 g of MBG nanoparticles was dispersed in 10 mL of CS solution (1 mg/mL) and stirred vigorously. The mixture was then sealed and incubated at 37 °C for 24 h under constant stirring to reach adsorption equilibrium. The resulting CS-loaded MBG nanoparticles were collected by high-speed centrifugation, and the supernatant was filtered through a 0.22 μm aqueous membrane to remove any suspended carrier residues. The absorbance of the supernatant was measured by UV-vis spectrophotometry, and the concentration of free CS was determined using a pre-established standard curve. The CS loading content was determined by an indirect method and calculated according to the following formula:

(1) $$DLC(\%)=\frac{m_{1}-m_{2}}{m_{0}}\times 100\%$$

Here, *m*_1_ denotes the total mass of CS added, *m*_2_ represents the mass of free CS, and m_0_ refers to the dry weight of the MBG carrier.

For the fabrication of the composite scaffold, 0.2 g of CS-MBG was added to a mixture containing 1 mL of Tris buffer (pH 8.0) and 10 mL of an aqueous dopamine hydrochloride solution (15 mg/mL). A pre-fabricated PLA scaffold was then immersed in this suspension. The system was stirred at room temperature for 10 h, allowing the formation of a PDA coating that incorporated CS-MBG onto the scaffold surface, thus yielding the final CS-MBG/PLA composite scaffold. For conciseness, CS-MBG/PLA is denoted as the CMP scaffold.

### 4.4. Preparation of Arg-CDs

Arg-CDs were synthesized via a hydrothermal method. Briefly, 3 g of L-arginine powder was dissolved in 25 mL of deionized water. The solution was transferred into a Teflon-lined autoclave and heated at 240 °C for 10 h. After filtration and dialysis using a dialysis bag (MWCO 500 D), the Arg-CD solution was collected. The purified Arg-CD solution was collected and lyophilized (Freeze Dryer SCIENTZ-25TK, Ningbo Scientz Freeze-Drying Equipment, Ningbo, China) to obtain Arg-CD powder for further use.

### 4.5. Synthesis of Gel-OSA/Arg-CD Hydrogel

OSA was first prepared as a crosslinking agent. Briefly, 4.28 g of NaIO_4_ was dissolved in 100 mL of deionized water, and 8 g of sodium alginate (SA) was dissolved in 200 mL of deionized water. The two solutions were mixed and stirred at room temperature in the dark for 6 h, with the molar ratio of sodium periodate to the repeating units of alginate set at 0.5. The oxidation reaction was terminated by adding ethylene glycol, followed by continued stirring for 1 h. The resulting suspension was dialyzed against deionized water (membrane cutoff: 3.5 kDa) for 72 h and subsequently lyophilized to afford OSA as a fluffy solid. The degree of oxidation—quantified via hydroxylamine hydrochloride titration—was determined to be 51.48%. For hydrogel formation, Gel, OSA, and Arg-CDs were combined under alkaline conditions using a 1% (*w*/*v*) sodium tetraborate (Na_2_B_4_O_7_) solution. The final concentrations in the hydrogel were 7.5% (*w*/*v*) Gel, 1.5% (*w*/*v*) OSA, and 1000 μg/mL Arg-CDs. The resulting composite hydrogel was designated GA. A control hydrogel without Arg-CDs was denoted GO.

### 4.6. Fabrication of the Gel-OSA/Arg-CDs/CS-MBG/PDA-PLA Composite Framework

The CMP scaffold (Section 4.3) was immersed in the precursor solution of the GA hydrogel (Section 4.5). After gelation at room temperature, the resulting composite was frozen at −20 °C for 12 h and then lyophilized to obtain the final hierarchical framework, designated as Gel-OSA/Arg-CDs/CS-MBG/PDA-PLA. For conciseness, this composite scaffold is hereafter abbreviated as GACP. A control scaffold prepared identically but without Arg-CDs (i.e., using the GO hydrogel) is denoted as GOCP.

### 4.7. Characterizations

The surface morphologies of the composite scaffolds were examined using scanning electron microscopy (SEM, MIRA, TESCAN, Brno, Czech Republic). Fourier-transform infrared (FT-IR) spectra of OSA, Gel, and the GO hydrogel were recorded on an FT-IR spectrometer (IR Prestige-21, Shimadzu Corporation, Kyoto, Japan) over the range of 400–4000 cm^−1^.

The morphologies of MBG and Arg-CDs were observed by transmission electron microscopy (TEM, FEI Talos F200X, Thermo Fisher Scientific, Waltham, MA, USA). The surface charges (zeta potential) of Arg-CDs were measured with a Zetasizer (Mastersizer Lab, Malvern Panalytical, Malvern, UK) after dispersing the samples in deionized water. The specific surface area and pore characteristics of MBG nanoparticles were determined by Brunauer–Emmett–Teller (BET) analysis (JW-BK200C, Beijing Jingwei Gaobo Instrument, Beijing, China). Arg-CDs were further characterized by ultraviolet–visible (UV-Vis) spectroscopy (UV-2450, Shimadzu Corporation, Kyoto, Japan). Particle size distributions of MBG and Arg-CDs were quantified based on TEM images using ImageJ software (ImageJ 1.54p).

The swelling behavior of the GA hydrogel was evaluated by immersing a pre-weighed freeze-dried sample in PBS at 37 °C. The sample was retrieved at predetermined time points, gently blotted, and weighed to calculate the swelling ratio. For in vitro degradation analysis, samples were incubated in PBS at 37 °C, retrieved at scheduled intervals, freeze-dried, and weighed. The degradation rate was calculated based on the remaining dry mass.

### 4.8. Release of the Arg-CDs and CS

To evaluate the release profiles of CS and Arg-CDs from the composite scaffolds, in vitro release studies were conducted in PBS, pH 7.4 at 37 °C under constant shaking (100 rpm). Specifically, samples containing 0.02 g of CS-MBG particles or corresponding CMP were immersed in 30 mL of PBS. For the release study of Arg-CDs, 500 μL of lyophilized hydrogel loaded with Arg-CDs was immersed in 4 mL of PBS, pH 7.4 under gentle agitation at 37 °C. At predetermined time points, the entire release medium was withdrawn and replaced with an equal volume of fresh pre-warmed PBS to maintain sink conditions. The concentration of Arg-CDs in the collected supernatant was quantified by UV-Vis spectroscopy at λ = 260 nm, using a calibration curve established with standard Arg-CD solutions. The cumulative release of both CS and Arg-CDs was monitored within the scheduled times and calculated using the following standard equation:

(2) $$CR=\frac{C_{n}\times V_{0}+\sum C_{n-1}\times V}{m_{0}}\times 100\%$$

where CR is the cumulative release, C_n_ denotes the concentration of Arg-CDs or CS measured in the sample solution collected at the nth sampling point, V represents the volume of liquid withdrawn at each sampling, m_0_ stands for the total amount of loaded Arg-CDs or CS, and V_0_ is the total volume of the release medium.

### 4.9. Antibacterial Ability of the Composite Scaffold

*Escherichia coli* (*E. coli*) and *Staphylococcus aureus* (*S. aureus*) were cultured in LB liquid medium at 37 °C with shaking at 100 rpm. Bacterial suspensions were adjusted to the required concentrations using sterile PBS for subsequent experiments. The MIC of Arg-CDs against *E. coli* and *S. aureus* was determined using the broth microdilution method in a 96-well plate. Arg-CD solutions were serially diluted in LB medium to final concentrations ranging from 15.7 to 1000 µg/mL. Each well was inoculated with a bacterial suspension to achieve a final density of 5 × 10^5^ CFU/mL. One column of the plate served as a bacterial growth control (without Arg-CDs), and another column served as a sterile medium control. After incubation at 37 °C for 15 h, the MIC was recorded as the lowest concentration of Arg-CDs that completely inhibited visible bacterial growth [35]. Prior to antibacterial assays, all scaffolds were sterilized by soaking in 75% ethanol for 30 min, followed by UV irradiation for an additional 30 min, and then thoroughly washed with sterile PBS to remove any residual ethanol.

Sterilized scaffolds (n = 3 per group) were immersed in 1 mL of bacterial suspension (1 × 10^6^ CFU/mL in PBS) and co-cultured for 24 h at 37 °C. After co-culture, 100 µL aliquots of the suspension were spread onto LB agar plates and incubated at 37 °C for 10 h for colony counting. Antibacterial activity was also quantified turbidimetrically. The co-cultured suspensions were transferred to a 96-well plate, and the optical density at 600 nm (OD_600_) was measured using a microplate reader (Epoch, BioTek Instruments, Logan, UT, USA). The bacteriostatic rate was calculated according to the following equation:

(3) $$Bacteriostatic\;rate(\%)=\frac{{OD}_{c}-{OD}_{S}}{{OD}_{C}}\times 100\%$$

where OD_C_ is the optical density of the control group suspension, and OD_S_ is the optical density of the sample-containing suspension.

To evaluate the effect of Arg-CD concentration on antibacterial efficacy, a well diffusion assay was performed. LB agar plates were uniformly inoculated with 100 µL of bacterial suspension (10^6^–10^7^ CFU/mL). Wells (6 mm diameter) were created in the agar, into which 100 µL of Arg-CD solutions at varying concentrations was added. After incubation at 37 °C for 18 h, the diameter of the inhibition zone (clear area around each well) was measured to assess antibacterial activity.

### 4.10. In Vitro Cell Culture

MC3T3-E1 pre-osteoblasts were maintained in α-MEM supplemented with 10% fetal bovine serum (FBS) and 1% penicillin–streptomycin. RAW264.7 macrophages were cultured in DMEM supplemented with 10% FBS and 1% penicillin–streptomycin. Both cell lines were incubated at 37 °C in a humidified atmosphere containing 5% CO_2_, with the medium refreshed every 2–3 days.

### 4.11. Biocompatibility of the Composite Scaffold In Vitro

Scaffold extracts were prepared by incubating sterile composite scaffolds in complete culture medium (α-MEM for MC3T3-E1, DMEM for RAW264.7) at 37 °C for 24 h. The supernatant was collected as 100% (*v*/*v*) extract. Cell proliferation was evaluated on days 1, 3, and 7 using a CCK-8 assay according to the manufacturer’s protocol. Absorbance was measured at 450 nm using a multimode microplate reader. For cytoskeletal observation, cells were cultured on coverslips in extract-containing medium for 24 and 72 h, fixed, and stained with Actin-Tracker™ Red-594. Fluorescence images were captured using a confocal laser scanning microscope (LSM880, Carl Zeiss AG, Oberkochen, Germany).

### 4.12. Cell Osteogenic Differentiation In Vitro

Scaffolds were incubated in osteogenic induction medium (OIM) to prepare osteogenic extracts. MC3T3-E1 cells were then cultured in these extracts for 7 and 21 days to assess differentiation. After 7 days, ALP activity was evaluated. Cells were fixed and stained using a BCIP/NBT Alkaline Phosphatase Color Development Kit, and ALP activity was quantified using a commercial ALP assay kit. Results were normalized to total cellular protein content determined with a BCA protein assay kit. Mineralization was assessed after 21 days by Alizarin Red S (ARS) staining, with quantification achieved through 10% cetylpyridinium chloride extraction. All staining results were observed under an optical microscope (NCM series, Novel Optics, Ningbo, China).

### 4.13. Anti-Inflammatory Activity In Vitro

To evaluate the scaffold’s anti-inflammatory activity in vitro, RAW264.7 cells were seeded into 96-well plates and maintained in complete DMEM. After 24 h, the medium was replaced with complete DMEM containing 1 μg/mL LPS to induce inflammation. Following an additional 24 h incubation, inflammation-induced cells were treated with the gel extract for 24 h. Subsequently, secreted inflammatory cytokines (IL-10, IL-6, IL-1β, TNF-α) were quantified using ELISA kits. Parallel cultures were assessed for intracellular ROS production using a detection kit, with visualization performed by confocal microscopy. For flow cytometry analysis, inflammation-induced RAW264.7 cells were cultured in 6-well plates following the same protocol and treated with scaffold extracts. Cells were then harvested, and intracellular ROS levels were quantified using flow cytometry (Attune NxT, Thermo Fisher Scientific Inc., Waltham, MA, USA).

### 4.14. Statistical Analysis

All quantitative data are presented as mean ± standard deviation (SD). Unless otherwise specified, experiments were performed with three independent replicates (n = 3). For comparisons involving more than two groups, a one-way analysis of variance (ANOVA) was performed, followed by Tukey’s post hoc test for pairwise multiple comparisons. Statistical significance was defined as *p* < 0.05. Asterisks indicate the levels of significance: * *p* < 0.05, ** *p* < 0.01, *** *p* < 0.001. All experiments were performed in triplicate to ensure reproducibility and reliability.

## Data Availability

The original contributions presented in the study are included in the article. Further inquiries can be directed to the corresponding author.

## Supplementary Materials

The following supporting information can be downloaded at https://www.mdpi.com/article/10.3390/gels12060471/s1, Figure S1: Results of ROS levels and cytokine secretion at day 5: (a) Quantification of ROS levels in RAW264.7 cells as measured by the fluorescence intensity of DCFH-DA. (b) Flow cytometry analysis of the scale of ROS-positive cells. (c–f) The levels of IL-6, IL-10, IL-1β and TNF-α in the supernatant of RAW264.7 cells after various treatments.

## References

[1] Mathieu L., Mourtialon R., Durand M., Rousiers A., I’Escalopier N., Collombet J.M. (2022). Masquelet technique in military practice: Specificities and future directions for combat-related bone defect reconstruction. Mil. Med. Res..

[2] Tian Y.Z., Sun R.L., Li Y.F., Liu P., Fan B., Xue Y. (2024). Research progress on the application of magnesium phosphate bone cement in bone defect repair: A review. Bio-Med. Mater. Eng..

[3] Ball J.R., Shelby T., Hernandez F., Mayfield C.K., Lieberman J.R. (2023). Delivery of Growth Factors to Enhance Bone Repair. Bioengineering.

[4] Chen Y.Q., Hu W.H., Dong Z.C., Dong S.W. (2022). Multi-functional osteoclasts in matrix-based tissue engineering bone. Chin. J. Traumatol..

[5] Li Y., Li X., Zhu L., Liu T., Huang L. (2025). Chitosan-based biomaterials for bone tissue engineering. Int. J. Biol. Macromol..

[6] Wang Y.L., Zhang H., Hu Y., Jing Y.Y., Geng Z., Su J.C. (2022). Bone Repair Biomaterials: A Perspective from Immunomodulatory. Adv. Funct. Mater..

[7] Cai W.Y., Xu X.P., Jiang Y.C., Cheng K., Liu F., Song C., Guo D.R., Hu Z.M., Liu Z.H., Liu Z.C. (2023). Programmed release of hydrogel microspheres via regulating the immune microenvironment to promotes bone repair. Mater. Today Adv..

[8] Vlachopoulos A., Karlioti G., Balla E., Daniilidis V., Kalamas T., Stefanidou M., Bikiaris N.D., Christodoulou E., Koumentakou I., Karavas E. (2022). Poly(Lactic Acid)-Based Microparticles for Drug Delivery Applications: An Overview of Recent Advances. Pharmaceutics.

[9] Rocha C.R., Chávez-Flores D., Zuverza-Mena N., Duarte A., Rocha-Gutiérrez B.A., Zaragoza-Contreras E.A., Flores-Gallardo S. (2020). Surface organo-modification of hydroxyapatites to improvePLA/HAcompatibility. J. Appl. Polym. Sci..

[10] Tang P., Song P., Peng Z., Zhang B., Gui X., Wang Y., Liao X., Chen Z., Zhang Z., Fan Y. (2021). Chondrocyte-laden GelMA hydrogel combined with 3D printed PLA scaffolds for auricle regeneration. Mater. Sci. Eng. C.

[11] Machado E.A.M., Rocha A.C.D., de Menezes L.R. (2025). Applicability of Hydrogels as Platforms for Bone Regeneration: A Mini-Review. Polym. Adv. Technol..

[12] Shaygani H., Mofrad Y.M., Demneh S.M.R., Hafezi S., Almasi-Jaf A., Shamloo A. (2024). Cartilage and bone injectable hydrogels: A review of injectability methods and treatment strategies for repair in tissue engineering. Int. J. Biol. Macromol..

[13] He R.Z., Gu Y.L., Jia J.Y., Yang F., Wu P., Feng P., Shuai C.J. (2025). Semiconductor photocatalytic antibacterial materials and their application for bone infection treatment. Nanoscale Horiz..

[14] Kunnath A.P., Suoodh M.S., Chellappan D.K., Chellian J., Palaniveloo K. (2024). Bacterial Persister Cells and Development of Antibiotic Resistance in Chronic Infections: An Update. Br. J. Biomed. Sci..

[15] Guo L., Kong W., Che Y.L., Liu C., Zhang S.C., Liu H.S., Tang Y.X., Yang X., Zhang J.Z., Xu C.N. (2024). Research progress on antibacterial applications of metal-organic frameworks and their biomacromolecule composites. Int. J. Biol. Macromol..

[16] Wu J., Lei J.H., Li M., Zhang A., Li Y., Liang X., de Souza S.C., Yuan Z., Wang C., Chen G. (2024). Carbon Dots Crosslinked Egg White Hydrogel for Tissue Engineering. Adv. Sci..

[17] Sheng L., Wang Z., Song L., Yang X., Ye Y., Sun J., Ji J., Geng S., Ning D., Zhang Y. (2024). Antimicrobial carbon dots/pectin-based hydrogel for promoting healing processes in multidrug-resistant bacteria-infected wounds. Int. J. Biol. Macromol..

[18] Meziani M.J., Dong X.L., Zhu L., Jones L.P., LeCroy G.E., Yang F., Wang S.Y., Wang P., Zhao Y.P., Yang L.J. (2016). Visible-Light-Activated Bactericidal Functions of Carbon “Quantum” Dots. ACS Appl. Mater. Interfaces.

[19] Bing W., Sun H.J., Yan Z.Q., Ren J.S., Qu X.G. (2016). Programmed Bacteria Death Induced by Carbon Dots with Different Surface Charge. Small.

[20] Zhang H., He J., Xiong Y.Y., Mu H.X., Deng Y.Q., Zhao Q. (2023). Antibacterial mechanism analysis and structural design of amino acid-based carbon dots. J. Mater. Sci..

[21] Li P.L., Han F.X., Cao W.W., Zhang G.K., Li J.Y., Zhou J.W., Gong X.D., Turnbull G., Shu W.M., Xia L.G. (2020). Carbon quantum dots derived from lysine and arginine simultaneously scavenge bacteria and promote tissue repair. Appl. Mater. Today.

[22] Pant S., Thomas S., Loganathan S., Valapa R.B. (2022). 3D bioprinted poly(lactic acid)/mesoporous bioactive glass based biomimetic scaffold with rapid apatite crystallization and in-vitro Cytocompatability for bone tissue engineering. Int. J. Biol. Macromol..

[23] Álvarez-Carrasco F., Varela P., Sarabia-Vallejos M.A., García-Herrera C., Saavedra M., Zapata P.A., Zárate-Triviño D., Martínez J.J., Canales D.A. (2024). Development of Bioactive Hybrid Poly(lactic acid)/Poly(methyl methacrylate) (PLA/PMMA) Electrospun Fibers Functionalized with Bioglass Nanoparticles for Bone Tissue Engineering Applications. Int. J. Mol. Sci..

[24] Gritsch L., Perrin E., Chenal J.M., Fredholm Y., Maçon A.L., Chevalier J., Boccaccini A.R. (2021). Combining bioresorbable polyesters and bioactive glasses: Orthopedic applications of composite implants and bone tissue engineering scaffolds. Appl. Mater. Today.

[25] Liu Y., Ma Y., Zhang J., Xie Q., Wang Z., Yu S., Yuan Y., Liu C. (2017). MBG-Modified β-TCP Scaffold Promotes Mesenchymal Stem Cells Adhesion and Osteogenic Differentiation via a FAK/MAPK Signaling Pathway. ACS Appl. Mater. Interfaces.

[26] Hoppe A., Güldal N.S., Boccaccini A.R. (2011). A review of the biological response to ionic dissolution products from bioactive glasses and glass-ceramics. Biomaterials.

[27] Guo K., Wang Y., Feng Z.X., Lin X.Y., Wu Z.R., Zhong X.C., Zhuang Z.M., Zhang T., Chen J., Tan W.Q. (2024). Recent Development and Applications of Polydopamine in Tissue Repair and Regeneration Biomaterials. Int. J. Nanomed..

[28] Yu J., Huang X., Chen X.H., Hu P.Y., Liu T., Zhang T.T., Cheng R., Cui T.T., Li J. (2024). Antibacterial and anti-inflammatory Bi-functional carbon dots hydrogel dressing for robust promotion of wound healing. Carbon.

[29] Yang X., Li P., Tang W., Du S., Yu M., Lu H., Tan H., Xing X. (2020). A facile injectable carbon dot/oxidative polysaccharide hydrogel with potent self-healing and high antibacterial activity. Carbohydr. Polym..

[30] Li P., Liu S., Cao W., Zhang G., Yang X., Gong X., Xing X. (2020). Low-toxicity carbon quantum dots derived from gentamicin sulfate to combat antibiotic resistance and eradicate mature biofilms. Chem. Commun..

[31] Navarro G., Gómez-Autet M., Morales P., Rebassa J.B., Llinas Del Torrent C., Jagerovic N., Pardo L., Franco R. (2024). Homodimerization of CB2 cannabinoid receptor triggered by a bivalent ligand enhances cellular signaling. Pharmacol. Res..

[32] Li J., Ma J., Sun H., Yu M., Wang H., Meng Q., Li Z., Liu D., Bai J., Liu G. (2023). Transformation of arginine into zero-dimensional nanomaterial endows the material with antibacterial and osteoinductive activity. Sci. Adv..

[33] Ye P., Yang Y., Qu Y., Yang W., Tan J., Zhang C., Sun D., Zhang J., Zhao W., Guo S. (2024). LL-37 and bisphosphonate co-delivery 3D-scaffold with antimicrobial and antiresorptive activities for bone regeneration. Int. J. Biol. Macromol..

[34] Sun X., Lin H., Zhang C., Huang R., Liu Y., Zhang G., Di S. (2022). Improved Osseointegration of Selective Laser Melting Titanium Implants with Unique Dual Micro/Nano-Scale Surface Topography. Materials.

[35] Sadaqat M.H., Mobarez A.M., Nikkhah M. (2022). Curcumin carbon dots inhibit biofilm formation and expression of esp and gelE genes of Enterococcus faecium. Microb. Pathog..

