# Supplementary material for: Carbon Dot-Linked Hydrogel-Composite Scaffold with Sequential Release of Multi-Drug for Bone Repair
Source: Gels. 2026 May 29;12(6):471. doi: 10.3390/gels12060471 (PMC13298461; doi:10.3390/gels12060471)
Supplement: Supplementary file 1 [file gels-12-00471-s001.zip › gels-4278460-supplementary.pdf]

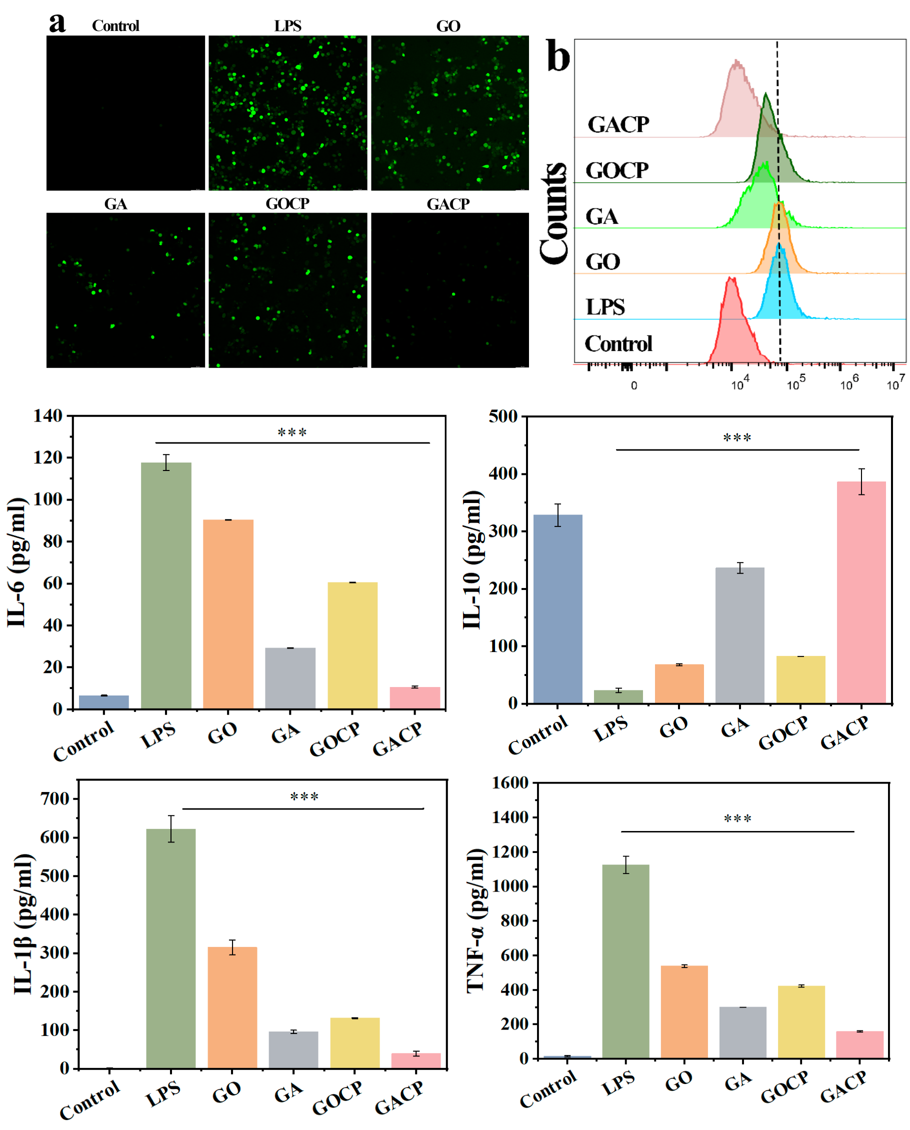

Fig.S1 Results of ROS levels and cytokine secretion at day 5: (a) Quantification of ROS levels in RAW264.7 cells as measured by the fluorescence intensity of DCFH-DA. (b) Flow cytometry analysis of the scale of ROS-positive cells. (c-f) The levels of IL-6, IL-10, IL-1β and TNF-α in the supernatant of RAW264.7 cells after various treatments.
